# Bioengineering of Artificial Lymphoid Organs

**Published:** 2016

**Authors:** M. A. Nosenko, M. S. Drutskaya, M. M. Moisenovich, S. A. Nedospasov

**Affiliations:** Engelhardt Institute of Molecular Biology, Russian Academy of Sciences, Vavilova str. 32, 119991, Moscow, Russia; Lomonosov Moscow State University, Faculty of Biology, Leninskie Gory 1, bldg. 12, 119991, Moscow, Russia; Deutsches Rheuma-Forschungszentrum (DRFZ), Charitéplatz 1, 10117, Berlin, Germany

**Keywords:** artificial lymphoid organ, bioengineering, polymeric scaffold, stromal cells

## Abstract

This review addresses the issue of bioengineering of artificial lymphoid organs.Progress in this field may help to better understand the nature of the structure-function relations that exist in immune organs. Artifical lymphoid organs may also be advantageous in the therapy or correction of immunodefficiencies, autoimmune diseases, and cancer. The structural organization, development, and function of lymphoid tissue are analyzed with a focus on the role of intercellular contacts and on the cytokine signaling pathways regulating these processes. We describe various polymeric materials, as scaffolds, for artificial tissue engineering. Finally, published studies in which artificial lymphoid organs were generated are reviewed and possible future directions in the field are discussed.

## INTRODUCTION

In recent years, bioengineering technologies have attracted a lot of attention, because they provide new approaches to resolving current theoretical and practical issues in biology and medicine. The development of artificial biocompatible materials opens up broad prospects for regenerative medicine, transplantation, treatment of infectious diseases and cancer, as well as for fundamental studies of a number of important aspects of tissue organization in living organisms, which require preservation of the spatial structure of the object under study. A fairly wide range of biocompatible and non-toxic biotechnological materials have been developed that can maintain the functions of different cells in three dimensional space. Furthermore, these biomaterials, particularly scaffolds or matrices, which will be discussed later, can be “functionalized” for a particular task. This has served as a stepping stone for the development of artificial organs and tissues based on polymer scaffolds, including artificial bones [1-4], skin [5, 6], cardiac tissue [7], and other tissues and organs. The potential development of functional artificial lymphoid organs, mainly secondary or tertiary ones, e.g. lymph nodes and lymphoid follicles, attracts particular attention [8-10], because such structures can in theory be used for the correction of immunodeficient states and for the treatment of autoimmune and infectious diseases and malignant neoplasms. It is assumed that bioengineered immune organs will be partially or completely responsible for the protective function in a human body with underlying pathological conditions [10]. Functional artificial secondary lymphoid organs (e.g. artificial lymph nodes) will make it possible to study and model some as-of-now poorly understood aspects of the immune response, and in the future they may find application in the immunotherapy of a whole range of diseases. An important difference between new immunomodulation approaches and the current systemic techniques (e.g., systemic cytokine or anti-cytokine therapy, depletion of lymphocyte populations, etc.) is the fact that the former act on the level of recognition of specific antigens by the immune system and will, therefore, minimize the negative impact of systemic immunotherapy and focus primarily on the cause of the disease. Their advantage over classical vaccination lies in the creation and long-term maintenance of the most favorable microenvironment, which enables all the key cellular interactions involved in the immune response to take place. In many cases, this can be the decisive factor for the success of a therapy [11, 12].

## STRUCTURE OF LYMPHOID ORGANS AND THEIR ROLE IN THE IMMUNE RESPONSE

Lymphoid organs are integral structural parts of the immune system, and disorders that affect them can result in immunodeficiency in humans and animals [13, 14]. There are three groups of organs: primary, secondary, and tertiary. In a normal adult organism, primary and secondary lymphoid organs are present at all times, whereas tertiary organs are generated locally at the site of a strong and sustained immune response: for example, at the site of a tumor or chronic inflammation [15]. Primary lymphoid organs – thymus and bone marrow – generate immune cells and define the repertoire of T- and B-lymphocytes receptors, whereas secondary and tertiary organs ensure their survival, interaction with other cells, interplay between innate and adaptive immune responses, as well as activation and maintenance of the immune response. Therefore, modeling of various lymphoid organs will help to resolve a variety of issues both in fundamental science and in medicine.

The functionality of immune organs relies on their unique microarchitecture and the wide range of cells and factors involved. Therefore, the challenge of bioengineering is to reproduce them in model systems, since functional activity can only be achieved through proper organization of all components. It is essential to understand the mechanisms of cell organization in the natural organs of a body in order to be able to construct their artificial analogues.

All immune organs are characterized by the presence of the stroma, which often consists of several types of cells of endothelial, mesenchymal, and, in some cases, epidermal origin [16, 17]. The main functions of the stroma include the recruiting and spatial organization of immune cells in the organ, maintenance of their viability, proliferation, and the enabling of effective interaction with other cells and antigens. Each organ has a type of stroma that is necessary for its functioning. The bone marrow of an adult organism produces all hematopoietic cells, including all types of leukocytes, from hematopoietic stem cells (HSC) and hematopoietic progenitor cells. The bone marrow maintains the HSC population via special niches that ensure long-term HSC repopulation, their differentiation into hematopoietic progenitor cells, and the generation of all necessary germs of differentiation [18, 19]. In addition, bone marrow, via certain bone marrow stromal cells, plays an important role in the differentiation and functionalization of B-lymphocytes, memory cells, plasma, and other immune cells [20].

Many types of hematopoietic cells completely, or almost completely, mature within the bone marrow. However, progenitors of T-lymphocytes must undergo several further stages of maturation in the thymus. The stroma of the thymus, i.e. thymic epithelial cells (TEC), enables the survival and selection of thymocytes, and different TEC subpopulations implement both positive and negative selection [21]. The key role in negative selection belongs to stroma-associated dendritic cells that actively present autoantigens [22]. Another important component of the thymus is the mesenchymal compartment responsible for the functioning of both epithelial and hematopoietic cells. Numerous interactions between hematopoietic, mesenchymal, and epithelial cells play an important role in all these processes [23]. The stroma facilitates the release of “trained” mature T-lymphocytes from the thymus, which, in the context of MHC molecules, can recognize the widest repertoire of foreign antigens while simultaneously being the least aggressive against autoantigens [16, 24].

In lymph nodes, the white pulp of the spleen and other secondary and tertiary lymphoid organs, the stromal cells recruit mature immune cells and enable antigen presentation and activation of T- and B-lymphocytes, which leads to their further differentiation, proliferation, and implementation of their effector functions, as well as the formation of memory cells [25-27]. Lymphoid organs associated with the intestines (mesenteric lymph nodes, Peyer’s patches, isolated lymphoid follicles, and cryptopatches) play a special role [28]. They are involved in the regulation of the relationship between host and symbiotic microflora, the development of tolerance to non-pathogenic bacteria and food-borne antigens, and the response to pathogenic microorganisms [29-31].

**Fig. 1:**
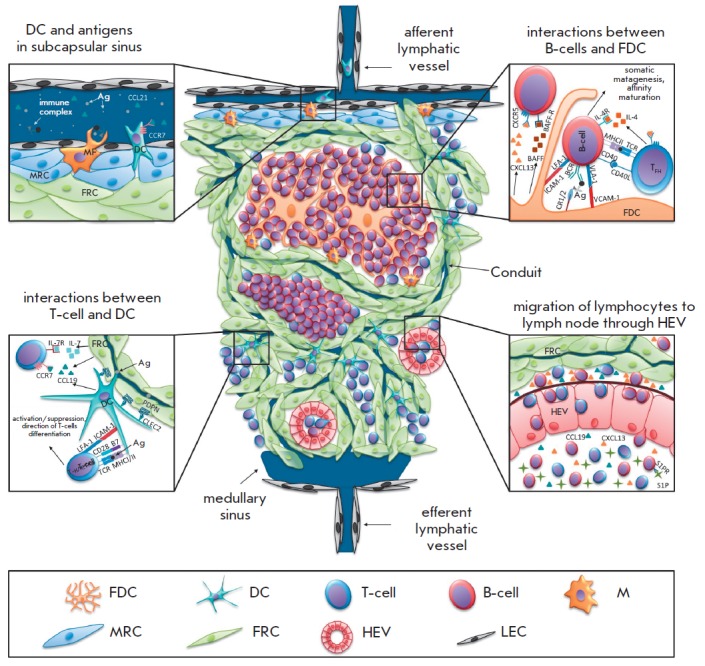
Schematic representation of a lymph node structure. The key events are shown in detail. Lymph from afferent lymphatic vessels enters the organ through the conduit system. It is then collected in the medullary sinus from which efferent lymphatic vessels originate. Soluble antigens, immune complexes, and antigen-presenting DCs enter LN with the afferent lymph. Other hematopoetic cells enter LN with blood vessels through HEV in response to attracting chemokines (CCL19, CXCL13 and others). All these cells then become distributed between the T- and B-zones of the organ. Egress of lymphocytes from LN is controlled by S1P that is produced by endothelial cells outside the organ. Stromal cells play an important role in regulating all these processes. They produce all necessary factors, cytokines, chemokines, adhesion molecules, and form the appropriate organ structure to support the functions of immune cells

Secondary and tertiary immune organs are of particular importance in the bioengineering of artificial lymphoid organs, since they play the central role in the initiation of the adaptive immune response [26], and, therefore, the processes occurring in them are of great interest for fundamental research and as the basis for potential clinical interventions in different pathological conditions. Therefore, the development and the structure of these organs will be discussed in more detail using lymph nodes as an example. The structure of a typical lymph node and the major steps of the adaptive immune response within it are presented in *Fig. 1*.

Anatomically, lymph nodes are bean-shaped encapsulated organs connected to the circulatory and lymphatic systems by a network of vessels. Two major groups of lymph nodes are identified based on their location in the body: mesenteric ones that are involved in the immune response and development of antigen tolerance in the intestines, and peripheral ones that collect the lymph from various regions of the body, primarily from barrier tissues. This distinction is based not only on their anatomical localization, but also on their functional differences, as these two groups of organs have different origins and functions [32-35]. Cervical lymph nodes hold a special place among peripheral lymph nodes due to the nature of their development during embryogenesis and their involvement in mucosal immunity [36, 37]. Despite the differences in their origin and functions, the anatomic structure of all lymph nodes is rather similar. They have two main sections: the cortex, forming the main parenchyma of the organ, and the medulla, which communicates with the efferent lymphatic vessels carrying the lymph from the organ [38]. The area of the cortex bordering the medulla is called paracortex. On the outside, a lymph node is enclosed in a capsule through which the organ communicates with the afferent lymphatic vessels. Connective septa (trabecula) originates from the capsule and goes inside the organ, up to the medullary sinus that forms the lymph node gate [39]. The area between the capsule and the cortex is called the subcapsular space.

The blood vessels are connected to the organ through the gate; then they go into the paracortex, which is also called the T-zone, where a network of capillaries is formed. Lymphoid follicles, also called B-zones, are located in the cortex of the lymph node [38]. The name of the zones corresponds to the location and function of these two major groups of lymphocytes in a lymph node, although it does not reflect many details of cell migration and interactions, which have only been discovered in recent years (thanks to the development of techniques that allow for the intravital imaging of individual cells in tissues and organs [40]). B-lymphocytes primarily function in the B-zone; whereas T-cells are generally located in the paracortex, except for follicular helper lymphocytes, which play an important role in the functioning of B-lymphocytes [41]. The presence of separate B- and T-zones in lymph nodes is possible due to the development of special microenvironments within them, which produce both lymphocyte survival factors and “homeostatic” chemokines (for example, BAFF and CXCL13 cytokines are the key factors for Bzones; and IL-7, CCL21, CCL19 are the key factors for T-zones) [25, 42-44]. These molecules are synthesized mostly by lymph nodes stromal cells, as well as by other cell types, including endothelial and dendritic cells [42]. B-zones contain follicular dendritic cells that are involved in the maturation of B-lymphocytes, which are of mesenchymal origin [45], whereas T-zones have dendritic cells of hematopoietic origin, which are involved in antigen presentation to T-lymphocytes [46]. Dendritic cells of hematopoietic origin mostly arrive with the afferent lymph from different areas of the body, mainly from the barrier tissues where they have encountered antigens, have been activated, and have begun to express the CCR7 chemokine receptor that is responsible for their migration into lymph nodes Tzones. There are also resident lymph node dendritic cells, which are always present in an organ [47]. Their role is to present antigens absorbed directly from the lymph flowing into the lymph node through a special system of channels, called conduits. These channels are formed by an extensive network of polymers, including collagen I, II, IV, laminin, fibronectin, ER-TR7 *et al*. [48].

lymphocytes are constantly re-circulated in the body, periodically being recruited into different lymph nodes under the influence of chemokines. The appearance of these cells in the lymph node is very important for homeostasis of the immune system, as lymph nodes stromal cells are the main source of survival factors for mature lymphocytes [42]. The time which a lymphocyte spends in a lymphoid organ is defined by the balance of chemotactic signals. Once a lymphocyte enters the lymph node paracortex via special high endothelial venule cells under the influence of the “homeostatic” chemokine concentration gradient, the expression of the sphingosine-1-phosphate (S1P) receptor in the lymphocyte gradually increases. The concentration of this factor in the blood and lymph is very high, but its production in lymph nodes is almost absent [49]. Under the influence of the gradient of S1P concentration, lymphocytes arrive in the medulla and subsequently egress through the efferent lymphatic vessels into lymph circulation. The interaction of the receptor with its ligand S1P results in the internalization of the complex and disruption of the chemotactic signal, allowing the cells to regain their ability to penetrate lymph nodes under the influence of the gradient of chemokine concentration in the blood [50]. This system enables efficient re-circulation of lymphocytes in the body, which is necessary for the selection of lymphocytes with the optimal specificity of T- and B-cell receptors (TCR and BCR) for the antigens at the time present in the body [51].

In addition to recruiting and maintaining the homeostasis of immune cells, lymph nodes also enable all the interactions necessary for an effective immune response, which is mediated not only by the properties of antigen-presenting and effector cells, but also by the spacial architecture of a lymph node [26]. For example, the cortex is permeated by a system of conduits, which has optimally arranged antigen-presenting cells and through which the lymphocytes move. This spatial arrangement provides the best chance for these two types of cells to meet, which facilitates the search for receptors specific for a particular antigen presented on dendritic or other antigen-presenting cells, among the vast repertoire of T-cell receptors [8, 48, 51].

## LYMPH NODES STROMAL CELLS

The contribution of individual types of stromal cells to the maintenance and functioning of the lymph nodes, their interactions, and origins remain poorly understood. To date, the most studied mesenchymal stromal cells of the secondary lymphoid organs are fibroblast reticular cells (FRCs) and follicular dendritic cells (FDCs) [33, 40, 43, 50, *Fig. 1*]. The former are primarily involved in T-lymphocytes functioning, whereas FDCs are necessary for full functionality of B-zones [25, 42]. FRCs form and maintain a system of conduits required for the migration and interaction of immune cells and delivery of antigens from the lymph [48, 52]. Three main types of endothelial cells are essential for the functioning of a lymph node: lymphatic endothelial cells (LEC), blood endothelial cells (BEC), and their variant, high endothelial venules cells (HEVC) [33, 42]. The role of these cells is to maintain constant contact between the node and the lymphatic and circulatory systems, or, more precisely, to ensure the exchange of immune cells and antigens. LECs ensure the recruitment and penetration of migratory dendritic cells into a lymph node, as well as the transfer of antigens from the lymph to the system of conduits inside lymph nodes [53, 54]. Conventional BECs line the blood vessels inside the node, whereas HEVC facilitate lymphocyte migration from the blood into the lymph node paracortex whence they are distributed to the respective zones of the node [42]. Recently, another type of stromal cells has been discovered which is located in the subcapsular zone of the lymph nodes and are present in other secondary lymphoid organs, but they are absent from tertiary ones; they are called marginal zone reticular cells (MRCs) [55, 56]. It has been shown that MRCs are the immediate precursors of FDCs, including being involved in the formation of germinal centers in follicles [57]. It has also been suggested that they play a role in maintaining the FDCs pool, but this requires further evidence.

The main obstacle in this field of research is the lack of consensus in the proper definition of different stromal cell types; despite fairly comprehensive characterization of the stromal cells functions, their exact phenotype is still a matter of debate and different authors adhere to different points of view [33, 42, 45, 58]. This can partly be attributed to the fact that only some stromal cells have universal surface markers. Many surface molecules are non-specific markers present in many cell populations. For example, the adhesion molecules ICAM-1 and VCAM-1 are considered to be the major markers for most of the mature lymph nodes stromal cells and their progenitors; these molecules enable both intercellular contacts in the stroma and interaction with incoming immune cells that express appropriate integrins on their surface [32, 59]. A glycoprotein podoplanin (gp38) is an important marker for some types of lymph node stromal cells, primarily LECs and FRCs. This molecule plays an important role in maintaining a normal state of vascular endothelial cells and a lymph node capsule, regulates the supply of blood and lymph to the node, migration of dendritic cells, and adaptive FRC reaction in case of a strong inflammation [54, 60]. All endothelial cells express CD31 as the primary endothelial marker. Specific surface markers are unknown for most lymph nodes stromal cells, and they are classified either by a combination of several “pan-markers” or based on the expression of specific genes and production of respective factors; however, this classification has not been fully accepted yet. For example, until recently the expression of CXCL13 chemokine in a mature lymph node had been attributed solely to FDCs, putative key players in the B-cell response. However, there is now evidence that marginal zone reticular cells (MRCs) and even FRCs can also synthesize CXCL13, and disruption of its production by these cells has a significant impact on the function of B-lymphocytes and the immune response [42, 44]. Nevertheless, a portion of stromal cells can be identified by the expression of a combination of several surface markers. For example, FDCs express CD35, CD21 (complement receptors), FcγRIIB, which detect immune complexes for subsequent presentation to B-lymphocytes in the germinal centers, and do not express typical hematopoietic surface markers (e.g., CD45) [45]. MRCs and, possibly, FDCs express MAdCAM-1 adhesion molecules [55]. FRCs are often identified on the basis of production of extracellular matrix components, which are necessary for conduits assembly, e.g. ER-TR7 [58]; however, these markers can only be used in immunohistochemical staining of lymph nodes sections, but not in cytometry when the cells are not bound to the matrix components. LECs typically express the Lyve-1 marker, and in mature lymph node HEVCs, in contrast to BECs, specifically express PNAd adressin and MAdCAM-1 adhesion molecules [42].

To summarize, the stromal compartment of lymph nodes and other secondary lymphoid organs is under active investigation and many aspects still have to be elucidated to fully understand the functions of all participating cells. This insight is important for the bioengineering of artificial lymphoid organs, the purpose of which is to create a functional organ from a minimum number of well-characterized components. The data on the functioning of lymphoid organs suggest that in order to be effective an artificial lymph node must have the appropriate infrastructure, which is mainly represented by a properly organized stromal compartment. The presence of all the necessary components of the stroma will define the effectiveness of a particular immune response that occurs in the system, and it will allow one to track the development of an artificial organ based on the analysis of the stromal cells’ composition.

## EMBRYONIC DEVELOPMENT OF LYMPH NODES

**Fig. 2:**
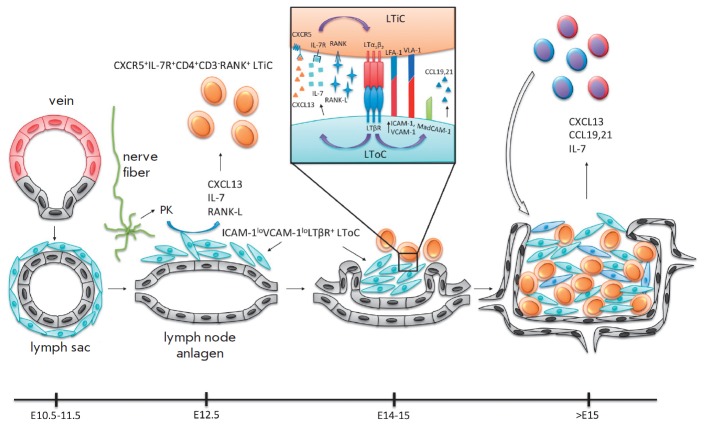
Schematic representation of mouse lymph node development. It begins with lymph sacs formation from venule endotelial cells. These sacs then produce the entire lymphatic system and some of them produce lymph nodes. Initiation of lymph node development seems to be dependent on retinoic acid (RA) production by proximal nerve fibers. RA probably signals through its receptor on mesenchymal cells around lymph sacs and stimulates them to produce CXCL13 chemokine and other cytokines. CXCL13 promotes migration of LTiC to the lymph sac, leading to the formation of lymph node anlagen. LTiC express on their surface LTαβ that interacts with LTβR on mesenchymal cells, converting them to LToC. This process is key in the development of most of the secondary lymphoid organs, including the lymph nodes. Activation of LTβR leads to further differentiation of LToC from which all mature lymph node stromal cell subtypes seem to originate. LToC produce many LN-specific chemokines and cytokines. They also start to express adhesion molecules on their surface, such as MAdCAM-1 and PNAd, which are required for the migration of lymphocytes to the lymph nodes. Lymphocytes act on stromal cells in many ways, engaging different members of the TNF superfamily, like TNF itself , LT, LIGHT and others. This promotes further maturation of the lymph node stromal cells and the formation of T- and B-zones.

Successful bioengineering of artificial lymph nodes requires good understanding of the processes that define the development of lymphoid organs during the embryogenesis. Such knowledge may allow one to differentiate all necessary types of cells from their progenitor cells extracted from fetal tissue or make it possible to develop the whole organ from the progenitor cells. Lymphoid organ development is shown in *Fig. 2*, using a lymph node as an example. It has been established that during the embryogenesis a lymph node anlagen is created in certain areas as a result of venules endothelial cells differentiation into lymphatic endothelium [61] and the formation of an endothelial lymph sac, which later participates in the development of the capsule and network of conduits in the lymph node and connects the organ to the lymphatic and circulatory systems [32]. Further development involves poorly differentiated mesenchymal cells around the vessels (pericytes), which are progenitors of FDCs, and, apparently, of all other stromal cells except for endothelial ones [45]. This has recently been demonstrated for the development of the spleen: FDCs, FRCs, and other stromal cells were derived from progenitor cells which expressed the transcription factors Nkx2-5 and Islet-1, important for the embryogenesis of spleen and pancreas [62]; however, for lymph nodes the origin of all types of stromal cells from a single population of progenitor cells requires more rigorous evidence. Lymph sacs originate the lymphatic system of the body, as well as lymph nodes. Lymph node location is defined by local secretion of retinoic acid (RA) by nerve fibers endings [63]. Under the influence of RA, mesenchymal progenitors begin secreting CXCL13 chemokine, which attracts lymphoid-tissue-inducer cells (LTiC), with the adhesion molecules ICAM-1 and VCAM-1 on their surface. From that moment onward, the mesenchymal progenitor cells are called lymphoid-tissue-organizer cells (LToC). LTiCs migrate to the lymph node anlagen, primarily under the influence of the CXCL13 chemokine concentration gradient and interact with LToC [63]. It has been established that signal transmission through LTβR located on the LToC surface is crucial at this stage [64]. The LTα1β2 heterotrimer is the main LTβR ligand involved in the lymph node embryogenesis, and it appears on the LTiCs surface after their interaction with a soluble factor, TRANCE (RANK-L), the exact source of which is unknown, but it is assumed that LTiCs can themselves be the source [59, 65, 66]. Mice deficient in LTβR or LTα have no secondary lymphoid organs (except for the nasal-associated lymphoid tissue [67]), and mice with genetic LTβ inactivation develop only cervical and mesenteric lymph nodes, which suggests that this signaling pathway is critically important for embryonic development [34, 59]. This signal pathway triggers further LToCs differentiation, which leads to increased expression of adhesion molecules and appearance of MAdCAM-1 and PNAd on the cell surface, as well as to increased expression of chemokines that attract new hematopoietic cells to the location of the future lymph node [32, 59, 64]. Signaling through the TNFR1 receptor is another important molecular cascade for the development of lymphoid organs. It has been demonstrated that genetic inactivation of either TNF or TNFR1 in mice leads to disruption of FDCs development and, consequently, to the absence of germinal centers in lymphoid organs [68]. It should be noted that members of the TNF superfamily play an important role in the development and maintenance of not only lymph nodes, but also all other lymphoid organs [34, 59, 65, 68-71]. Therefore, there is a synergy of different signaling pathways, which eventually results in a fully developed and functional immune system.

The next step of development, apparently, involves the accumulation of hematopoietic cells in the forming lymph node, which results in its growth, further differentiation of stromal cells, development of high endothelial venules, primordial follicles, and other compartments characteristic of lymph nodes [32, 59, 72]. At the initial stages, the development of structural compartments in lymph nodes does not involve Tor B-lymphocytes; however, at the later stages they actively penetrate into the organs and participate in the final maturation of lymphoid follicles and further maintenance of the stroma infrastructure via LTβR and TNFR signaling [26, 36]. In addition to LTα1β2, another LTβR ligand, LIGHT, plays a crucial role in this process [65]. Thus, the development and functioning of a mature lymph node (as well as other secondary lymphoid organs) depends strongly on the interaction between mesenchymal and hematopoietic cells, which should be taken into account in the bioengineering of these organs. Both cell components (as mature cells or, possibly, progenitor cells) must be properly arranged in the lymph node development site for its effective maturation and subsequent functioning.

## BIOMATERIALS FOR ARTIFICIAL ORGANS ENGINEERING

In addition to the minimum set of cell types required for the functioning of an artificial lymph node, it is important to create a framework that will serve as a structural scaffold for a proper spatial arrangement of the cells, a prerequisite for their effective interaction. During ontogenesis, the stromal cells create the necessary structure themselves and it consists primarily of polymeric, preferably collagen, fibers [58, 59]. In the case of bioengineering of an artificial lymph node, it is necessary to initially create a three-dimensional scaffold on which the cells will create a three-dimensional cell culture, and, subsequently, a fully fledged node. This is extremely important at the early stages, when the cells have not yet created their own polymeric scaffold necessary for further differentiation, survival, and functionality.

**Fig. 3:**
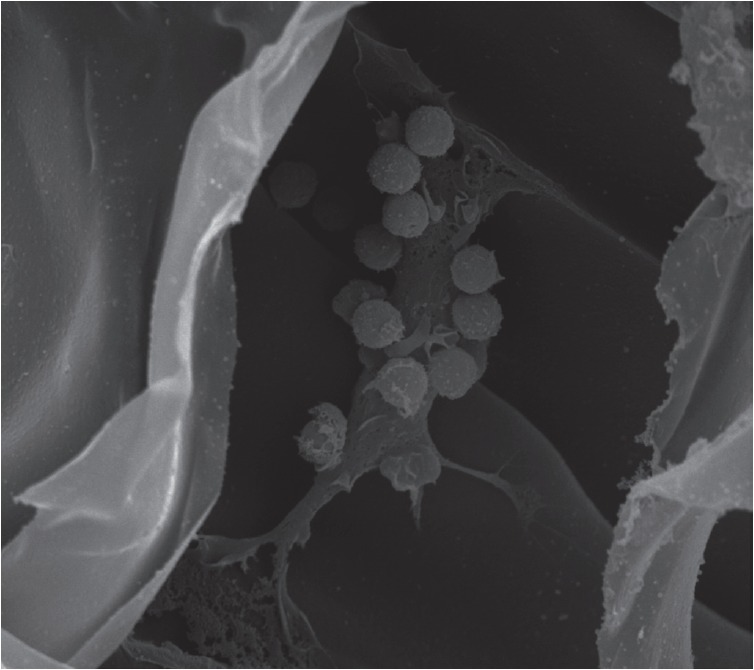
Polymeric fibroin scaffolds for artificial lymphoid organ bioengineering. A SEM image of a mixed culture of bone marrow-derived dendritic cells and splenocytes is shown. A dendritic cell (in the middle) interacts with a group of lymphocytes (around the DC) on the surface of the scaffold seen on the periphery

Artificial scaffolds based on biomaterials appear to be the most promising ones for the bioengineering of lymphoid organs. Such materials consist primarily of modified polymers of natural origin, both polysaccharides and proteins: fibroin (main component of the silk cocoon of silkworm *Bombyx mori*) (*Fig. 3*) [35, 36], spidroin (primary component of a spider’s web) [73-75], alginate (mixture of polysaccharides from algae cell wall) [76], collagen [77], etc. Synthetic polymers are also used, e.g., PLG (polylactate-co-glycolate), PLA (polylactate), PGA (polyglycolate), etc [78]. Various modifications of the substrate (e.g. by hydroxyapatite or collagen (gelatin)) are used to improve certain polymer properties such as elasticity, immunogenicity, adhesiveness, and resistance to outside factors [79].

The absence of antigenic, carcinogenic, toxic, and other properties limiting their use in medicine is a mandatory requirement for bioengineering materials. Such effects are typically associated with the presence of reactive groups formed by monomers or initiators of a polymerization reaction in the polymer substrate. Therefore, for future use it is necessary to carefully monitor the composition of the material and its purification and modifications [80]. Adverse effects *in vivo* are the main reason why many biomaterials have not yet found wide clinical application. There have been attempts to improve the properties of bioengineered materials; several types of structures which have been shown to be biocompatible in animal experiments have already been selected [11, 75]. However, it is impossible to avoid the immune response entirely, since most materials are allogenic. The main objective in the development of these materials is to avoid a systemic response and acute inflammation.

All these biomaterials usually come in the form of three-dimensional scaffolds with a cellular structure which is as close as possible to the fibrous structure of the extracellular matrix in animal tissues. By analogy, such bioengineered structures are also referred to as matrices, emphasizing their use as scaffolds; three-dimensional backbones for the cultivation of plated cell populations. This approach has been applied primarily in the preparation of 3D cell cultures, since it is known that many features of cell interaction in a functioning organ cannot be reproduced *in vitro *on plate surfaces [81]. The matrix may be a gel-like polymer network, for example, a collagen matrix, or it can have a more solid structure which retains its shape during mechanical manipulations, such as implantation into an animal model. The latter is the most important for the potential use of the material in medicine, direct studies of cell behavior in living models, and for the bioengineering of artificial organs. A variety of approaches are used to create the appropriate shape and texture of the matrix [78]. Polymerization of a monomer which will form the matrix has to create the appropriate three-dimensional porous structure; otherwise, the scaffold space will not be available for colonization by cells or saturation by any other substance.

In some studies, natural extracellular matrices from animal organisms, freed from inhabiting cells, are used to improve biocompatibility [82]. These materials do not induce an immune response upon implantation, since they are not allogenic, but they are usually more susceptible to enzyme degradation, which can be both advantageous and undesirable, depending on the task at hand.

However, scaffolding for an artificial organ is not the only implementation for such systems. Recently, the use of scaffolds for binding various kinds of soluble biological factors and their subsequent gradual diffusion from the matrix have been attracting increasing attention, since it ensures a gradual and extended release of biologically active substances over time. In the context of the bioengineering of artificial lymph nodes, this approach is promising, because it allows one to create an artificial gradient of chemokines and growth factors which may be necessary for the initiation of the maintenance program for organ development. As discussed above, during embryogenesis cells are recruited to the lymph node site and subsequently differentiated via the expression of, primarily, CXCL13 chemokine by stromal cell progenitors. In constructing artificial organs, special polymer particles can become sources of this chemokine, and they are being developed by several research groups at the moment. For example, a group of authors [83] has reported on the development of alginate microspheres which can be saturated by a chemokine and will be subsequently gradually released into the medium. Such microspheres have a size of 5–20 •µm, which allows for various manipulations; e.g., they can be used as a source of substances that attract cells for organs and tissues modeling *in vitro*. Another approach to the development of materials that ensure a controlled release of factors is to use biodegradable polymers covalently crosslinked by an active substance. The action of the enzymes or spontaneous hydrolysis will also gradually release the factors linked to the matrix, allowing them to perform their biological function [84].

## ADVANCES IN BIOENGINEERING OF ARTIFICIAL LYMPHOID ORGANS

As we have noted, bioengineering of artificial organs is important, first and foremost, due to its potential clinical application. The possible use of these organs for reprogramming the immune response in a number of diseases is very appealing. For each specific task, the final system does not even have to have all the characteristics of normal organs, but rather only those that are relevant to the necessary function. If artificial lymph nodes are able to ensure recruitment, survival, interaction, activation and functioning of immune cells, it will be possible to direct the immune response towards the specific, most effective, direction. Artificial lymph nodes can be populated *in vitro *by activated DCs that are loaded with specific antigens. After implantation of such systems, dendritic cells will interact with incoming lymphocytes, regulating their differentiation and functional activity. The advantage of such systems over vaccination with antigens or administration of a suspension of activated DCs lies in the fact that they will represent the vast majority of antigen-presenting cells in an artificial lymph node, and therefore there is a high probability that every incoming lymphocyte, specific to the antigen, will be influenced by certain cytokines and costimulatory molecules at the DCs surface. The implantation of such systems directly into the center of tumor growth or autoimmune reactions is expected to allow one to reprogram the specific lymphocytes and, therefore, will afford therapeutic effect. Although the implementation of this idea still requires a lengthy process of development of a fully functional artificial lymph node, it is already possible to create truncated systems that may have some clinical significance. The major published advances in this field are summarized in the *Table.*

**Table:** Published models of artificial lymphoid organs

Type of object	Polymeric scaffold	Cellular composition	Functional activity	Reference
Bioengineered vaccine against melanoma	PLG-matrix saturated with GM-CSF, CpG and tumor cells lysate	Myeloid dendritic cells	Recruiting of dendritic cells, resulting in their maturation, and subsequent migration into the draining lymph node, activation of anti-tumor immune response	[11, 49, 51]
In vitro model of a human lymph node, a bioreactor	Polysulfone bioreactor with polypropylene fibers as vessels and agarose matrix on which the cells were grown	Myeloid dendritic cells, T- and B-lymphocytes	After the system is initiated: secretion of cytokines and specific antibodies, formation of immune memory in response to an antigenic stimulus	[10, 52]
FRCs compartment of a murine lymph node	Polyurethane matrix modified with collagen	Fibroblast reticular cells (FRCs)	Adaptive FRCs response to changes in the fluid flow rate through the matrix, including increased production of the CCL21 and CCL19 chemokines and elevated rate of cell division	[50]
Artificial murine lymph node	Collagen matrix	Thymic epithelial cell line which produced LTα, and myeloid dendritic cells	After implantation of the matrix with the cells under the kidney capsule: migration of lymphocytes, formation of T and B-zones, production of antigen-specific antibodies. After the transfer of the artificial lymph node into immunodeficient mice production of antibodies was restored	[53, 54]
Artificial murine thymus	No polymeric scaffold	Aggregates of fetal thymocytes, mesenchymal cells and Foxn1+ fibroblasts	After implantation into athymic mice: the aggregates ensured full thymic function, generated naive T-lymphocytes of all major subtypes	[55]

For example, the feasibility of using a matrix for vaccination against melanoma has been demonstrated in mice [11, 85]. For this purpose, a PLG-matrix was saturated with a granulocyte-macrophage colonystimulating factor (GM-CSF), a synthetic oligonucleotide containing unmethylated CpG repeats, and with partially lysed melanoma cells. The study measured the therapeutic effect on the melanoma. Previously, it has been established that GM-CSF is essential in recruiting and activating murine dendritic cells. CpG was added to stimulate DCs differentiation towards a direction leading to the activation of type I T-helper cells, which are considered to be the most appropriate response to a tumor [86]. It has been shown that implantation of such “structural vaccines” as a three-dimensional matrix leads to the recruitment of murine skin DCs, their activation, and subsequent migration into the draining lymph node. In the node, they are involved in the maturation of specific T-helper type I cells, which ultimately leads to an increased anti-tumor response in a mouse, which manifests itself as reduced mortality in transplanted tumor models. In this paper, the matrix, which was saturated with factors of differentiation, recruitment of dendritic cells, and tumor antigens, partially functioned as a tertiary lymphoid organ. This is the simplest possible model that, nevertheless, allowed functional interaction between the implanted structure and the mouse lymphatic system that resulted in a specifically directed immune response. It has been shown that tumors are rejected due to the induction of a strong cytotoxic response of CD8+ lymphocytes. Importantly, this treatment regimen has already been adapted to humans and is currently in the first stage of clinical trials (https://clinicaltrials.gov/show/ NCT01753089).

In another model, which was closer to a real immune organ [10], the authors tried to create a prototype of a human lymph node *in vitro*. To that end, they developed a bioreactor that imitated the organ’s position relative to the vascular system of the body. It consisted of a first chamber that contained the matrix with dendritic cells and represented a lymph node, and a second chamber that contained a suspension of lymphocytes to model blood flow. The chambers communicated with each other through a porous membrane that enabled free circulation of both the soluble factors and the cells. It has been shown that, subject to regular change of the medium, such a culture is quite stable and can survive for at least 2 weeks with preserved activity of the cells. After 2 weeks of cultivation, the matrix was found to contain T- and B-lymphocytes populations which came from the adjacent chamber of the bioreactor, in addition to the dendritic cells. Both the lymphocytes and dendritic cells formed clusters within the matrix, which could be an indication of their potential functional activity. This model was proposed as a possible test system for studying the effects of certain drugs, as well as for studying cellular interactions *in vitro*. The model is a rather faithful representation of some of the processes in a lymph node; namely, the migration of lymphocytes and their interaction with dendritic cells.

However, despite the advances in the bioengineering of truncated models of lymph nodes, it has become clear that a fully fledged organ cannot exist and function without its special constituent stroma. This resulted from both extensive studies of stromal cells biology and attempts to use them to model lymphoid tissue. These two lines of research were combined into one in [87]. A sufficiently strong local inflammation is known to results in increased size and cellularity of the draining lymph nodes. This adaptive reaction is supported by an elevated rate of FRCs divisions in the lymph node proximal to the inflammation site and increase in the level of expression of the CCL21 and CCL19 chemokines, which attracts a lot of lymphocytes and dendritic cells to this lymph node. Since one of the first changes in local inflammation is a significant increase in the lymph flow rate through the draining lymph node [88], it has been suggested that FRCs may react to changes in the lymph flow rate in the system of conduits, which initiates a number of functional changes, such as an increase in chemokines production by these cells. To test this hypothesis, the authors constructed an *in vitro* lymph node model consisting of a matrix populated with a stable line of fibroblast reticular cells, with controlled flow of the lymphatic fluid through the model. It was shown that the secretion of CCL21 and CCL19 by the cells was higher under fluid flow conditions than in the static system. Moreover, the lymph flow affected not only the expression of the chemokines, but also the rate of cell division, as well as their spatial organization in the matrix.

Remarkably, in the case of fluid flow, some cells formed specific channel-like structures oriented along the direction of the flow. This organization was not observed under the static conditions. In addition, the lymph flow resulted in a reorganization of the matrix by the cells located within it and creation of spatially oriented structures. Presumably, in addition to being involved in the lymph node response to inflammation, the lymph flow may also play a role in the organization of the organ structure, adjusting the position and function of the stromal cells.

This is a very interesting observation, made for a simple lymph node prototype consisting of just one type of stromal cells, which nonetheless clearly demonstrated a complex systemic interaction between all components that can be explored entirely in a model of an artificial lymphoid organ.

The biggest success in the bioengineering of artificial lymph nodes has been achiev by Japanese scientists, who have developed a system based on a collagen matrix [89, 90]. They populated these matrices with a TEL-2 thymic epithelial cell line [91] previously transfected with a vector containing a lymphotoxin α (LTα) gene, as well as DCs derived from a bone marrow culture. They implanted these structures under the kidney capsule of a mouse and observed the migration of recipient lymphocytes into the matrix and a spatial cluster-like organization of T- and B-cells in the matrix, similar to their organization in the lymph node. They have also demonstrated that prior population of the matrix with dendritic cells is necessary for efficient migration of recipient cells into it. Furthermore, cells expressing endothelial markers were detected in the matrix, which indicated blood vessels growth. After some time in a mouse model, the matrix was recovered and transferred into mice with severe, combined immunodeficiency (SCID). After transplantation, the immunodeficient mice displayed a migration of cells from the matrix into the spleen and secretion of IgG antibodies. If the populated matrices were initially implanted into mice previously immunized with a protein antigen (modified ovalbumin NP-OVA was used in the experiment), their subsequent transfer to immunodeficient mice resulted in the latter producing antibodies specific to that antigen. The wealth of data suggests that it is reasonable to call the system ‘an artificial lymph node’. It should be noted that this work used collagen matrices which quickly degraded and shrunk in size, which could have affected their efficiency in the long-term experiments. Bioengineered scaffolds should use more inert materials to ensure the long-term presence and functioning of an artificial lymphoid organ in a recipient’s body.

Development of artificial thymus is another equally important task. It may play an important role in medicine, since thymus evolution with age results in a decrease in the number of new T-lymphocytes in the human body and subsequent deterioration of the immune response to new infections. Development of artificial thymus could help solve this problem. Recently, a promising study [92] was published which describes a system for producing thymic epithelial cells (TECs), which are required for thymus functioning, using *in vitro *reprogramming of mouse embryonic fibroblasts (MEF) under the influence of Foxn1, a transcription factor important for TECs. It was shown that over the course of their differentiation the transformed cells acquired a normal TECs phenotype: they expressed surface markers (EpCAM) and genes for factors that are important for their functional activity (Dll4, CCL25, Kitl et al.). In addition, despite the involvement of the Foxn1 factor in the development of skin epithelial cells, the transformed MEFs do not express genes specific to them, which indicates their orientation towards thymic, rather than skin, epithelium. The resulting cells were then characterized by their ability to ensure *in vitro* maturation of T-cells progenitors, imitating the process that occurs in normal thymus. It has been shown that co-cultivation of a transformed MEF and T-cells progenitors leads to the formation of a transient population of thymocytes (CD4^+^CD8^+^), as well as terminally differentiated CD4^+^ and CD8^+^ naive T-cells in quantities comparable to those obtained when TECs isolated from embryonic mouse thymus are used as the stroma. Untransformed MEF did not enable thymocytes maturation. It is important that the resulting cells expressed MHC class II molecules at a level comparable with that of normal TECs. These MHC class II molecules were only expressed after T-lymphocytes progenitors were added to the culture, emphasizing the importance of interaction between stromal and hematopoietic cells for the functioning of the lymphoid tissues. It is well known that MHC class II molecules on the surface of TECs are important for the selection of thymocytes in the thymus by their ability to recognize major histocompatibility complex molecules and only insignificantly bound autoantigens in order to generate autotolerant functional cells. Notably, the latter function depends on the TECs *AIRE *gene that is expressed by *Foxn1*- transformed fibroblasts, as well. Finally, the resulting cells were used as a base for an artificial thymus model. For this purpose, tissue aggregates were produced from three cell types: T-lymphocyte progenitors, fetal thymus mesenchymal stroma as a source of survival factors, and transformed MEFs. Once these aggregates were produced, they were implanted under the mouse kidney capsule and thymic tissue formation was observed after 3–4 weeks. An examination of this tissue’s composition revealed that aggregates obtained using the transformed fibroblasts reproduced normal thymus tissue in terms of its structure and function. These organelles were comparable to the artificial tissue obtained by implantation of embryonic TECs, whereas cell aggregates with non-transformed MEFs were unable to produce thymus tissue. The resulting system can be confidently called a prototype of artificial thymus. Just like the normal thymus, it had two subtypes of TECs necessary for proper thymocytes selection and their spatial zoning. The expression profiles of TECspecific genes and surface markers were comparable to the profiles in the normal embryonic thymus. The artificial thymus was able to support T-lymphocytes differentiation towards both TCRαβ CD4^+^/CD8^+^ and TCRγδ T-lymphocytes. Finally, the implantation of the systems to athymic (nude) mice resulted in detection of mature naive T-lymphocytes in their peripheral blood and spleen, which confirms the full functionality of the organ. This work is an important step on the way towards bioengineering of artificial thymus, including for clinical purposes [92]. However, the issue of the mesenchymal compartment remained unaddressed, since in this work it was formed by the fetal thymus tissue, which would be impossible if the artificial organ is produced for an adult organism. Overcoming this obstacle will open the way to the development of fully functional bioengineered active organs that can promote better understanding of the nature of the thymocyte selection process occurring in the thymus and can become an important tool in the treatment of human immunodeficiency.

It should be stressed that while the approaches relying on transformed cell lines are useful for research, they have no potential clinical use. Moreover, even in the laboratory such models are limited to a single line of animals from which the culture is derived. Two possible approaches to resolving this issue have been proposed: the use of cell-free systems or primary cell cultures. The first idea is based on the introduction of a pre-defined mixture of factors into the matrix, which will be used as the basis for an artificial organ, in order to attract and ensure the survival and differentiation of lymphoid and stromal progenitor cells imitating normal lymph node development. Additional biomaterials ensuring a gradual release of substances, such as alginate microspheres, can be used to create a concentration gradient of these factors. Candidate factors include the CCL19, CXCL13 chemokines, the BAFF, IL-7, VEGF, PDGF cytokines, and others. Regulation of the dynamics of individual release for each factor can ensure recruitment of progenitor cells from the bloodstream and their subsequent differentiation, without prior colonization of the matrix by any cells. Recently, a model was described in which the matrix included two cytokines, VEGF and PDGF [93]. It has been shown that tailored release dynamics for each factor can effectively stimulate angiogenesis at the site of the matrix implantation, which is necessary for the migration of cells and nutrients into it. Construction of artificial organs using this approach will, of course, require a combination of a higher number of factors.

If a cell-free system proves not enough for the bioengineering of clinically efficient artificial lymphoid organs, it will become necessary to develop a protocol for the use of cells. In this case, the most promising approach appears to be associated with the use of induced pluripotent stem cells (iPSC), which are expected to be the “next big thing” in the field of personalized medicine [94]. To date, there are several published works in which iPSCs were successfully used to create models of human organs: e.g. small intestine [95, 96]. In the context of artificial lymphoid organs, iPSCs can serve as a source of stromal compartment which ensures functional activity of the organ. This possibility has recently been discussed in a review of artificial thymus bioengineering [97]. In the future, this promising approach can be applied to other lymphoid organs.

## CONCLUSION

Even though several models of artificial lymphoid organs have been developed to date, a critical analysis has identified several problems that still need addressing. The use of transformed cells places obvious limitations on clinical application. Organ bioengineering using primary cell cultures has also been of limited use, mostly due to the fact that there are no adequate experimental protocols for many types of cells, especially stromal cells, describing their isolation, cultivation, and maintenance of their differentiated state. The most promising approach for clinical use is to obtain all or most of the cell types either from their progenitors or by transdifferentiation of mature cells; e.g. via iPSCs. Some cell types are readily available in primary cultures, including those of human cells, and this has already become the basis of several therapy regimens, such as adoptive transfer of dendritic cells or lymphocytes. It is only natural to suggest a combination of the two approaches: introduce some cells directly into the artificial lymph node scaffold as primary cultures, and compensate for the lack of others by adding factors, anticipating that such a combination would be enough to initiate the process of organ formation, and that all other cells will develop there later from the recruited progenitor cells. Today, many studies are conducted in this field.

In summary, the development of artificial lymphoid organs is an important task in modern immunology and biomedicine both from the theoretical and practical points of view. Success in this area is associated not only with advances in bioengineering, but also with recent progress in understanding the processes of lymphoid organs formation and functioning. This subject is at the crossroads of several scientific fields: bioengineering, immunology, systems biology, and regenerative medicine and, therefore, requires a comprehensive approach to research which would combine different ideas and take into account all or most of the factors responsible for the functioning of such complex systems as lymphoid organs.

## Abbreviations

Ag: antigen
BEC: blood endothelial cells
DC: dendritic cells
FDC: follicular dendritic cells
FRC: fibroblastic reticular cells
HEVC: high endothelial venule cells
HSC: hematopoetic stem cells
iPSC: induced pluripotent stem cells
LEC: lymphatic endothelial cells
LTiC: lymphoid tissue inducer cells
LToC: lymphoid tissue orginizer cells
M: macrophages
MEF: mouse embryonic fibroblasts
MRC: marginal zone reticular cells
RA: retinoic acid
S1P: sphingosine-1-phosphate
TEC: thymus epithelial cells
TFH: T-follicular helper cells
